# A Novel Hybrid Foaming Method for Low-Pressure Microcellular Foam Production of Unfilled and Talc-Filled Copolymer Polypropylenes

**DOI:** 10.3390/polym11111896

**Published:** 2019-11-17

**Authors:** Gethin Llewelyn, Andrew Rees, Christian A. Griffiths, Martin Jacobi

**Affiliations:** 1 College of Engineering, Swansea University, Swansea, Wales SA1 8EN, UK; 2 Trexel GmbH, Ahlefelderstr. 64, D-51645 Gummersbach, Germany

**Keywords:** polypropylene, foam-injection molding, TecoCell^®^, MuCell^®^, talc, calcium carbonate

## Abstract

Unfilled and talc-filled Copolymer Polypropylene (PP) samples were produced through low-pressure foam-injection molding (FIM). The foaming stage of the process has been facilitated through a chemical blowing agent (C_6_H_7_NaO_7_ and CaCO_3_ mixture), a physical blowing agent (supercritical N_2_) and a novel hybrid foaming (combination of said chemical and physical foaming agents). Three weight-saving levels were produced with the varying foaming methods and compared to conventional injection molding. The unfilled PP foams produced through chemical blowing agent exhibited the strongest mechanical characteristics due to larger skin wall thicknesses, while the weakest were that of the talc-filled PP through the hybrid foaming technique. However, the hybrid foaming produced superior microcellular foams for both PPs due to calcium carbonate (CaCO_3_) enhancing the nucleation phase.

## 1. Introduction

The use of plastics has enabled “quick wins” for automotive manufacturers, along with other similar industry sectors, in terms of metal replacement. Ensuring that the use of polymers meets the growing legislative requirements and reduction in environmental damage requires the use of novel processing innovation. The manufacture of polymer components with a microcellular cross-section provides a novel solution that reduces component weight, material usage, and processing energy requirements [1,2,3,4,5]. Foam-injection molding (FIM) shows high potential in creating parts with higher dimensional accuracy with reduced final weight [6].

In comparison to conventional injection molding (IM), the benefits of using FIM components and the subsequent processing are witnessed in the weight reduction of the resulting parts which ranges from 0 to 15% [7]. Also, FIM produces parts with higher impact strength, higher energy absorption and better thermal and acoustic insulation [8].

The process of FIM is very similar to IM except that the packing phase can be either completely removed, or lowered considerably, depending on whether low-pressure or high-pressure FIM is being conducted, respectively [9,10]. The four additional steps for low-pressure FIM from IM are: gas mixing and dissolution within the polymer melt (occurs during the plasticizing stage), cell nucleation (during injection stage), growing of the cells and shaping within the mold (during polymer cooling) [11]. The benefits to using low-pressure FIM is that large component weight savings can be achieved. For low-pressure FIM the resulting component weight-saving is lower however the cell structure of high-pressure foaming shows better microcellular structure [12]. Towards nano-cellular microstructure has proven to improve thermal conductivity due to the Knudsen effect [13,14], improved relevant mechanical strength along with more consistent final parts [15].

Polypropylene (PP) is regarded as one of the most popular polymers used in polymer industry due to low material cost, excellent chemical resistance, low density and high heat distortion temperature [16]. The first stage of FIM is super critical fluid (SCF) dissolution into the polymer melt [17]. Semi-crystalline polymers, such as PP, exhibit poor SCF dissolution due to the special interference between crystalline lamella being smaller than the SCF molecules [11] which means that the single-phase solution is not formed correctly. This process can be improved by processing techniques: increasing the barrel pressure improves the SCF absorption but decreasing the barrel temperature has also shown to decrease SCF absorption [11].

Inorganic fillers, such as carbon black, silica, calcium carbonate, rubber, and talc, have been added to various PP structures in order to enhance the properties of the final part [18,19,20,21]. Fillers have also been shown to enhance the SCF dissolution and cell nucleation for semi-crystalline polymers [22,23]. When processing PP with the addition of fillers the mechanical properties of the resulting material can also be affected [7,11,24,25,26]. It has been previously observed that the skin formation structure ratio has a major influence on the mechanical properties of IM parts [27,28], as well as FIM parts [29]. This can be affected through isothermal crystallinity properties of various polymers, processing conditions and fillers [30,31,32]. The addition of talc improves the isothermal crystallization process of PP resulting in an improved nucleation affect [20].

Wang et al. researched the addition of talc into linear PP nanocomposite microcellular foamed parts [33]. The viscoelastic properties of the nanocomposites were improved, as well as the crystallization properties, with the talc addition, resulting in superior microcellular structures [33]. The presence of talc in PP resulted in a decrease of mobility of the polymer molecules, leading to an early crystallization and as a result a higher crystallization temperature [34]. Also, the addition of talc leads to an increase in cell concentration when used with a blowing agent [34]. Other studies have concluded that the gas dosage must be increased for unfilled PPs due to gas escape occurring due to low gas diffusion witnessed in the crystalline region [7,26,35].

FIM can be achieved via a chemical blowing agent (CBA) or physical blowing agent (PBA) [11]. Essentially, in both techniques the cellular structure formed via the blowing agent reduces the material cell density which in turn reduces the resulting polymer component weight [36]. When using PBA as the agent, (super critical nitrogen or carbon dioxide) it causes a contact action, whereas CBAs release inert gas upon heating, into the polymer [37]. Typical CBAs used are ammonium, sodium bicarbonate or very complex inert gas-releasing materials [34,38] and create a microcellular structure with larger cells and lower cell density, compared to PBAs [23].

The research presented in this article focuses on comparing various foaming methods of Copolymer Polypropylene components in IM through CBA, PBA, and, for the first time, a novel hybrid foaming method of both foaming agents in combination. First, FIM parts were produced with 1 wt %, 2 wt % and 5 wt % of CBA. Then, the 3 resulting parts were matched with PBA to produce 3 level of weight savings for both the unfilled and talc-filled PP. Finally, the two foaming methods were combined to again produce 3 weight savings; making 9 experimental settings for each PP. To assess the effectiveness of the hybrid foaming method, they were characterized by their tensile strength and flexural modulus. The internal skin thickness, cell density, and average cell diameters were assessed to draw conclusions from the research.

## 2. Materials and Methods

### 2.1. Materials

**Figure 1:**
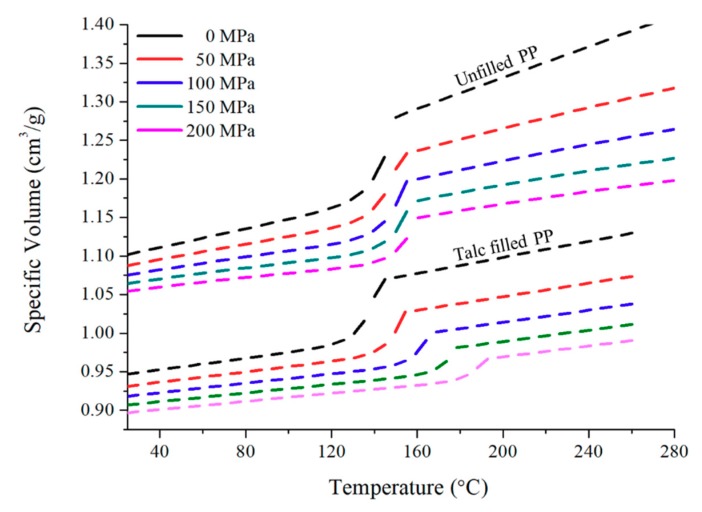
Pressure-Volume-Temperature data for unfilled PP and talc-filled PP.

Two commercial grades of Copolymer PP have been used for this research; unfilled PP and a 22% talc-filled PP, both of which are widely used in the automotive industry. Their melt flow rates (MFRs) are 15 and 35 g/10 min and they have densities of 905 and 1050 g/cm³, respectively. Figure 1 is the Pressure-Volume-Temperature (PvT) data for both polymers used in this research.

The CBA used was TecoCell^®^ H1 Chemical Blowing Agent, supplied by Trexel Inc. It creates an endothermic, chemical reaction at 200 °C between monosodium citrate (C_6_H_7_NaO_7_) and calcium carbonate (CaCO_3_) to release carbon dioxide (CO_2_) into the IM barrel [38].

### 2.2. Processing

Production of the injection molded parts were performed using an IM machine (e-Victory 120, ENGEL, Warwick, UK) with a clamping force of 1200 kN and a screw diameter of 40 mm. Alongside this IM machine, there is a gas dosing unit (T100, Trexel GmbH, Gummersbach, Germany) which enables MuCell^®^ IM to be performed. The SCF used for this research was nitrogen (N_2_) with 99.998% purity. Prior to any molding, the polymer was dried at 80 °C for a minimum of 4 hours in a commercial drier.

For both unfilled PP and talc-filled PP, the first processing performed was a conventional IM part. This was to get the benchmark figures to base the rest of the research from. After the conventional IM parts were produced, CBA was added to the process in increments of 1%, 2% and 5%. Prior to the experiment, preliminary experiments were conducted to ensure the appropriate stroke length was used based on the underpinning CBA content. For all the experiments performed on each polymer, everything was kept constant, and repeatability was performed by producing 50 parts for each experimental setting. The only changes made to the processing for each stage was the shot volume; this was lowered to accommodate for the extra foaming which was occurring.

The weight of the parts was taken and then the resulting weight was matched with the PBA. Here, the processing changes for each stage included the shot volume along with the SCF dosing amount. For higher weight reduction percentages; a higher SCF dosing was applied along with a compensation in the shot volume. For all the FIM processed in this paper, low pressure was used: using no holding pressure. This was only performed on the conventionally molded parts. The key processing data for each stage is listed in Table 1 for unfilled PP and Table 2 for talc-filled PP. The resulting average weight for each of these settings is also shown. All the other main processing parameters (Table 3) were used based on information from the material supplier recommendations and preliminary studies prior to these experiments.

**Figure 2:**
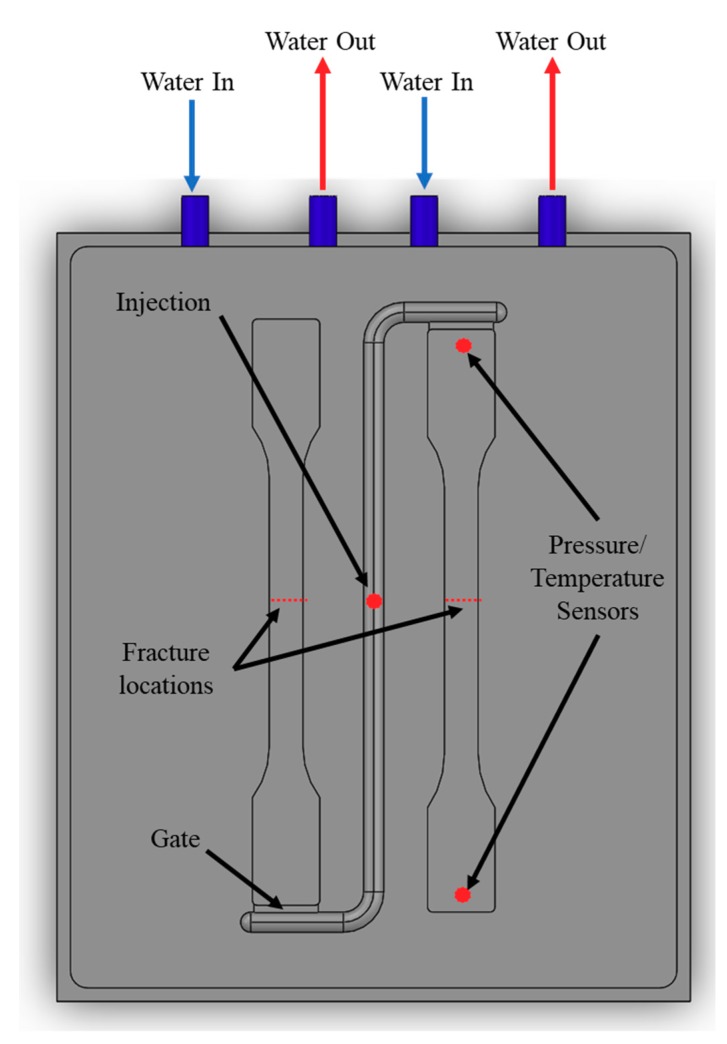
Mold setup with cooling channels and mold features including gate, injection and pressure/temperature sensor locations, and the region where samples were cryogenically prepared.

There are 2 tensile bars that are produced with each production, both complying to Type A1 within BS EN ISO 20753:2014 (Figure 2). The mold is made from Grade 6061 Aluminum.

### 2.3. Characterization

#### 2.3.1. Simple Modeling

Prior to any microscopy visualization or mechanical testing, simple modeling can be performed using the processing settings and simple model equations. These equations are listed below in further detail and have been verified in previously published research journals [39]. The model assumes that the cross-sectional structure of the foamed parts comprises of a foamed core with a skin surface, modeled by the integral skin foams (ISF) [39]. However, this is a simplified model as in reality, the cross-sectional structure varies throughout. There exist larger cells in the center, leading to smaller and better distributed cells nearer the skin and then a transition between the skin and this foamed center.

Enhancement of this modeling will be completed in further research but for this research, the model was used as a basic prediction to prove that it can provide prior knowledge to the experiments. The skin thickness was kept constant and as the samples used for this research were all semi-crystalline, a nominal value of 10% [39] of the average part geometry was used; in this case 0.7 mm.

Tensile strength model equations:

*R_tu_* = (*bh* − (*h* − 2*t*)(*b* − 2*t*) + (1 − *R*)^2^ (*h* − 2*t*)(*b* − 2*t*))/*bh* (1)

*R* = (*bh*(1 − *R_w_*))/((*h* − 2*t*)(*b* − 2*t*)) (2)

where *R_tu_* is the ratio of tensile strength of unfilled foamed polymer to unfilled solid polymer, *b* is the sample width, *h* is the sample thickness, *t* is the sample average skin thickness and *R* is the ratio of the weight reduction for the foamed core. All the parameters are known (and *t* is kept constant at 0.7 mm) and then *R* can be calculated using Equation (2) whereby *R_w_* is the weight reduction of the whole part.

Flexural strength model equations:

*R_fu_* = (2*t*^3^)/*h*^3^ + (6*t*(*h* − *t*)^2^)/*h*^3^ + (1 − *R*)^2^ ((*h* − 2*t*)/*h*)^3^ (3)

*R* = (*h*(1 − *R_w_*))/(*h* − 2*t*) (4)

Like the equations shown in the previous section, the flexural modulus of unfilled polymer shows proportionally to the square of the density when microcellular foamed. The flexural strength ratio, *R_fu_*, for unfilled polymer whereby all the inputs are the same as mentioned previously while the real reduction in the foamed core, R, can be calculated using Equation (4).

#### 2.3.2. Internal Cell Structure

The cell morphology of all the samples were examined at 85 mm along the part (midpoint). All parts were scorn in the required area and then submerged in liquid nitrogen for 4 hours before being snapped along the scorn line (Figure 2). These surfaces were analyzed using a scanning electron microscope (SEM) (Evo LS 25, Zeiss, Cambridge, UK). Low Vacuum mode was used to gain a clear contrast while also obtaining a large depth of view.

Gwyddion data visualization and data analysis software was used in order to measure skin thickness, cell density and average cell diameter [40]. The skin thickness was measured until the first cells were seen to be visible [29].

#### 2.3.3. Tensile Properties

The first of the mechanical properties analyzed on the samples was through tensile tests performed on a mechanical testing unit (H25 KS, Hounsfield, Surrey, UK). For each experimental setting performed, 10 parts were assessed. BS EN ISO 527-1:2012 was followed to obtain the maximum stress and an Axial Extensometer (3542, Epsilon, WY, USA), to calculate the Young’s Modulus. The maximum force was divided by the cross-sectional area to obtain the maximum stress (Equation (5)), while the chord slope method was used to calculate the tensile modulus between strain values of 0.0005 and 0.0025 (Equation (6)). The experiment was performed at 1 mm/min until a strain of 0.0025 had been met, then increased to 10 mm/min until break.

σ_M_ = F_M_/A (5)

*E* = dσ/dε (6)

#### 2.3.4. Flexural Modulus Properties

The other mechanical property that was analyzed was the flexural modulus of the samples. Again, 10 samples of each experimental setting were tested using a mechanical testing unit (H25 KS, Hounsfield, Surrey, UK). Due to the high flexural nature of the polymers, BS EN ISO 14125:2011 was followed with a 3 point flexural structure to accommodate for the high deflection. The large deflection correction calculation was used (Equation (7)) as all the deflections performed in this research complied to the criteria of the deflection being greater a tenth of the span. 2 mm/min speed was performed until the maximum force had been met and started to decrease.

σ_f_ = 3FL/(2bh^2^)[1 + 6(s/L)^2^ − 3((sh)/L^2^)] (7)

## 3. Results and Discussion

### 3.1. Simple Modeling Prediction

#### 3.1.1. Tensile Strength Comparisons

**Figure 3:**
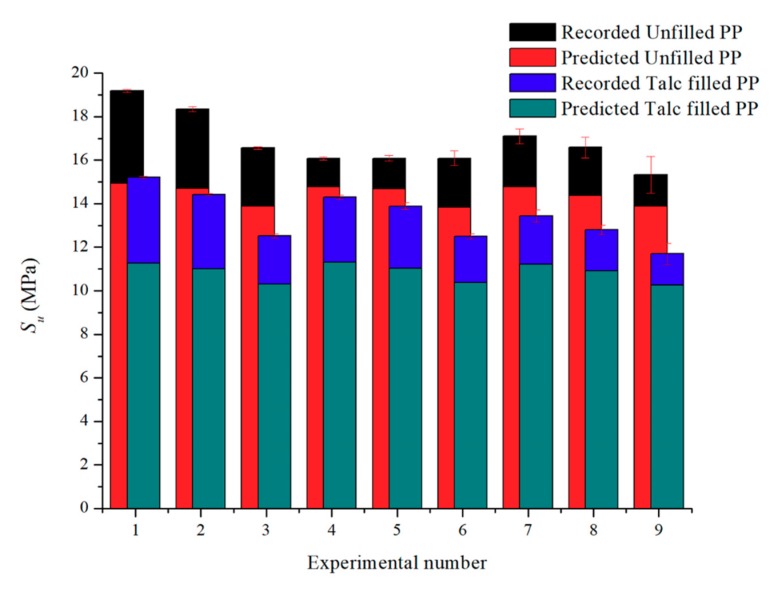
Recorded and predicted Ultimate Tensile Strengths for both polymers.

Figure 3 shows the tensile strength results against the predicted result through the simple modeling method for unfilled PP and talc-filled PP. The error range for unfilled PP is between 22.2% to 9.5% while the talc-filled PP has greater inaccuracies between 26.0% to 12.4%. The main reasoning of this is that the equations have not been modified to accommodate for the incorporation of talc into the polymer, the equations that were previously derived were for the addition of talc to a polymer and not for a polymer with talc already incorporated into its polymer matrix.

All the modeled results are underestimations of the actual results. From analyzing Equations (1) and (2), any minor changes to the *t* (thickness) value, results in a major difference in results. The skin thickness was averaged at 0.7 mm for all the samples in this research. However, Table 4 shows that the actual skin thickness varies from each experimental setting and thus, making the simple modeling a generalized prediction for this research. For both polymers, the models show greater accuracy in predictions for physical foaming and a combination of foaming methods and shows higher that 15% inaccuracies for all parts produced through chemical foaming.

#### 3.1.2. Flexural Strength Comparisons

**Figure 4:**
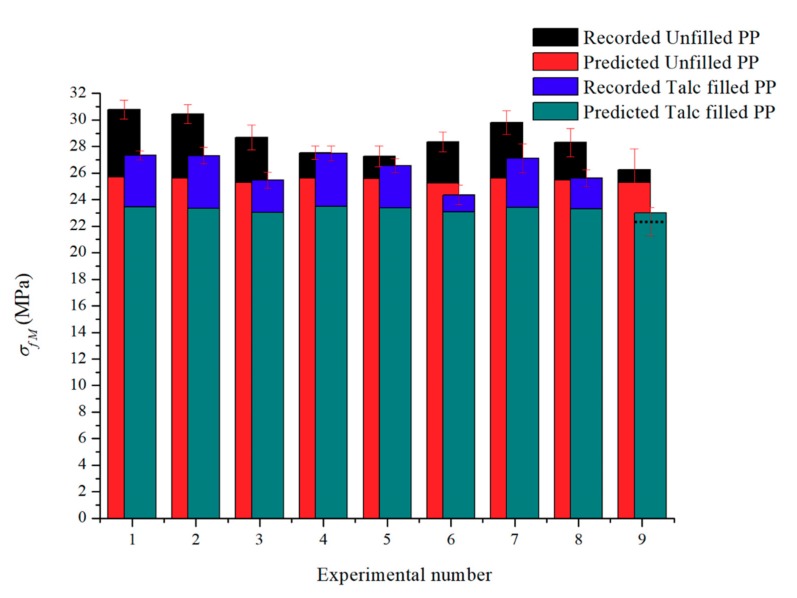
Recorded and predicted Maximum Flexural Strength for both polymers.

Figure 4 is the flexural strength results of the experimental and predicted for unfilled PP and talc-filled PP. The largest error is 16.5% while the smallest error is 3.1% (seen as the dashed line, whereby the predicted data for the talc PP is an over prediction). Thus, showing that the flexural strength simple modeling gives improved results compared to that of the tensile strength for the polymers in this experiment. As with the tensile results, the least accurate comparisons come from the chemical foaming agent while the most accurate is that of the hybrid foaming method.

### 3.2. Microscopy Characterization

#### 3.2.1. Unfilled PP Analysis

**Figure 5:**
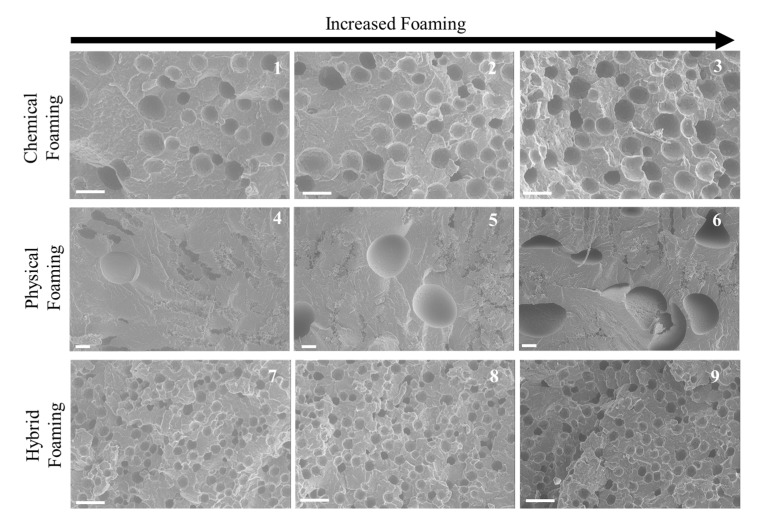
SEM images of unfilled PP cross-section (white bar indicates 100 µm).

As seen in Figure 5, the PBA settings gives the poorest quality cellular structure from all the experimental settings. The CBA gives some improved cellular structure but there are areas of no foaming within the part (excluding the skin structure). Meanwhile, the hybrid foamed parts gave the best cellular structure, this being confirmed in Table 4 as the smallest cell diameter and highest cell density is seen in the hybrid foaming of the unfilled PP. It is well known that unfilled PP is a poor choice of polymers for the foaming process due to the lamella in the semi-crystalline structure restricting the diffusion and also, the nucleation of the foaming process [11]. This would explain why the PBA parts in this polymer have exhibited poor cellular structure. The large cells are formed due to high shear stress applied to the polymer from the nucleation, whereby cell coalescence is present and causes many smaller cells to form larger ones [41]. CaCO_3_ has previously been added to PP in batch processing foaming method and shown to decrease the average cell diameter, increase the cell density and show a more homogenous cell structure [42,43,44]. With the composition of the CBA being mainly CaCO_3_, then this has proven to show good cellular structure with the foaming process with FIM.

#### 3.2.2. Talc-Filled PP Analysis

**Figure 6:**
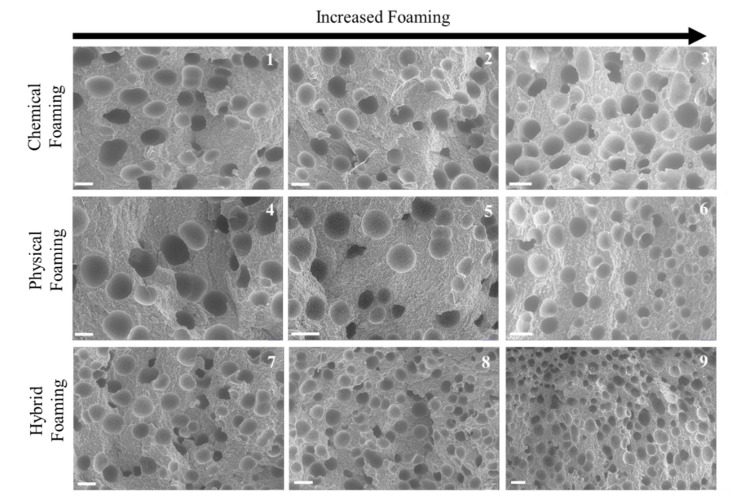
SEM images of talc-filled PP cross sections (white bar indicates 100 µm).

In comparison to the images for unfilled PP (Figure 5), it can clearly be identified that talc-filled PP shows enhanced properties for the foaming process (Figure 6), except for the previously explained hybrid foaming of the unfilled PP. The CBA still gives an improved cellular structure to that of the PBA, but the PBA is significantly better with the addition of talc. Like the unfilled PP, the hybrid foaming method gives superior microcellular structure for the talc-filled PP also, with the increased foaming producing smaller, finer cellular structure.

Table 4 confirms that the smallest cell diameters and highest cell density are indeed seen in the hybrid foaming of the talc-filled PP.

### 3.3. Tensile Strength Data

#### 3.3.1. Modulus of Elasticity (E)

For each experiment observed in Table 2 and Table 3, 10 parts were tested and the mean value of E and S_u_ were calculated. The effects of the selected process factors on the E of the parts were investigated together with the weight of the parts produced.

**Figure 7:**
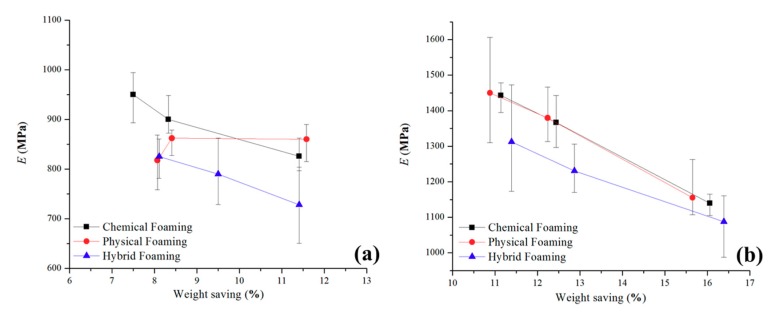
Young’s Modulus (E) against weight-saving (**a**) unfilled PP (**b**) talc-filled PP.

The mean value E plot for unfilled PP (Figure 7) shows that parts produced with chemical foaming have the highest ability to withstand changes in length when under tension, 949.7 MPa, this is then followed by the combination of the two foaming methods of 825.4 MPa. Physical foaming is the lowest with 817.5 MPa. For the parts made with chemical and the hybrid foaming the parts with a higher mass have an increased ability to withstand a change in length, both have a reduction of E with a decrease in the part weight. Interestingly, there is an increased deviation in E for the physical foam test parts with the lowest mass, which can be explained by the poor foaming ability of unfilled PP. Physical foaming results show a different stress strain behavior to chemical and the combination sample in that the E increases as the mass of the parts.

The talc-filled PP appears to show a more consistent behavior to that of the unfilled PP. The chemical and physical foaming follow similar pattern for the E values, with a decrease in weight: 1442.9 MPa, 1367.2 MPa and 1139.3 MPa for chemical foaming while 1450.3 MPa, 1379.6 MPa and 1155.1 MPa for the physical foaming. For the hybrid foaming method, the E values are lower with a reduction in weight at 1312.0 MPa, 1230.5 MPa and 1087.3 MPa. Like the unfilled PP, the parts produced through the hybrid foaming method, have a much larger deviation than the other two methods and thus make a more unpredictable part. The ratio of E and mass is also the highest for the hybrid foaming, coupled with the large deviation in these results identifies a significant material and foaming behavior in terms of the parts ability to withstand changes in length when under tension. The process control of physical foaming and PP talc results are more predictable with the inclusion of the talc filler when compare to the unfilled PP results. This can be justified that talc is a well-known nucleating agent for polyolefin materials which here, has shown to produce foamed parts with which have higher E values and are more consistent.

#### 3.3.2. Ultimate Tensile Strength (S_u_)

**Figure 8:**
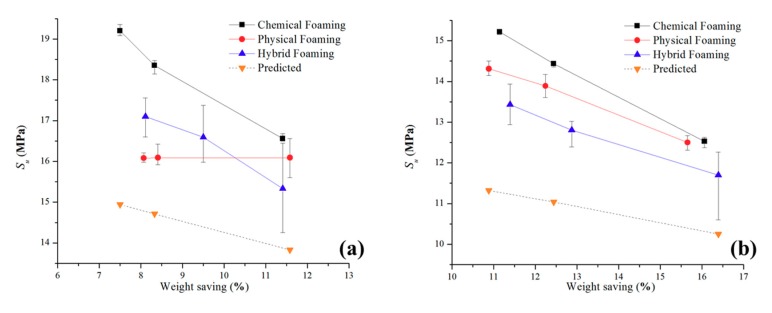
Ultimate Tensile Stress (S_u_) against weight-saving (**a**) unfilled PP (**b**) talc-filled PP.

Like the modulus of elasticity, the S_u_ of the parts were investigated together with the weight of the parts produced. The addition of the predicted results was also added to Figure 8.

The mean value S_u_ plot for unfilled PP (Figure 8a) shows that parts produced with chemical foaming have the highest ability to withstand loads that elongate the test part (19.2 MPa) this is then is then followed by the combination of the two foaming methods (17.1 MPa). Physical foaming is shown to be generally lower S_u_ and this is constant regardless of the mass of the test parts; as with the previous section, the poor results from the physical foaming of unfilled PP is well-known. All three foam processes demonstrate a tensile strength which is significantly higher than the simulated results. For the parts made with chemical and the combination of chemical and physical foaming the parts with the higher mass have an increased ability to withstand tensile loads. Like the E results, the hybrid foam methods have a much wider distribution of results. The deviation for the hybrid foaming methods is caused by the nucleation rate being too high, with two foaming methods combined, the nucleation energy is increased substantially and therefore causing large variations in morphology of the final part.

For the test parts produced with talc-filled PP (Figure 8b) the overall behavior is like the E results: in that the S_u_ reduces with reduced mass. The lower S_u_ values compared to the unfilled PP, can be explained as the ductility is hampered with talc addition due to stress concentrations and poor shear slipping ability of the polymer matrix [33]. S_u_ is a product of force per unit area and the non-homogeneous cross sections and bubble content that reduces mass clearly changes the test part unit area. Chemical and physical foaming methods have a higher S_u_ than the hybrid foaming method and all three are higher than the simulation results. The inclusion of a talc filler together with physical foaming has a notable influence on S_u_. There is a S_u_ decrease with a reduction in part mass; explained by the reduction in polymer across the sample’s cross-sectional area. However, for all combinations, the S_u_ is lower with the talc when compared to the unfilled PP. For both materials, the simply modeled results underestimate the S_u_. The likely cause to this is that the equations use a generic skin thickness and as seen in the microscopical characterization section from this research, this is not the case.

### 3.4. Flexural Modulus Data

**Figure 9:**
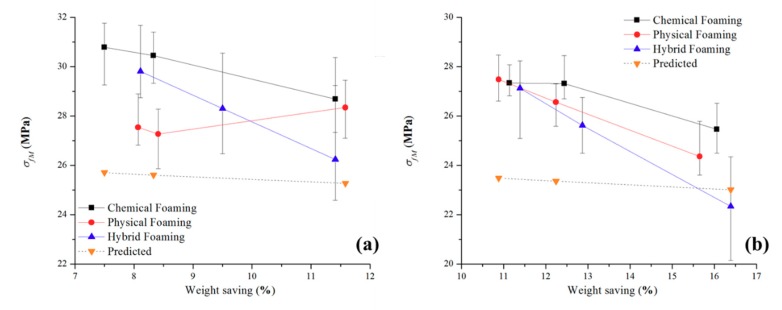
Maximum Flexural Strength (σ_fM_) against weight-saving (**a**) unfilled PP (**b**) talc-filled PP.

Tensile mechanical properties are important for polymer part design. In addition to E and S_u_ the mechanical measure of maximum load bearing capability of a material without undergoing any permanent deformation is considered. Maximum Flexural strength (σ_fM_) results are shown for the two PP materials (Figure 9).

The mean value σ_fM_ plot for unfilled PP (Figure 9a) shows that parts produced with chemical foaming have the highest ability to resist breakage when bent (30.8 MPa). This is then followed by the combination of the two foaming methods (29.8 MPa). For the parts made with chemical and the combination of chemical and physical foaming the parts with a higher mass can resist the highest stress. The values are 30.5 MPa and 28.9 MPa respectively, thus identifying chemical foaming not only as the highest σ_fM_ but also more consistent over a range of part masses. There is a wide range of deviation in σ_fM_ for the hybrid foam test parts and this is consistent for the tensile tests in the previous section. The physical foaming results show different σ_fM_ behavior when compared to the other foaming methods and this outcome is like the tensile results also.

The results for the test parts produced with talc-filled PP (Figure 9b) show a different behavior to the unfilled PP behavior. All foaming combinations have a lower σ_fM_ than unfilled PP. They also have a similar σ_fM_ (27.5–27.3 MPa) at high mass and this σ_fM_ decreases as the mass decreases due to more foaming in the parts. Again, chemical foaming is more consistent in terms of mass and mechanical properties. In terms of the parts ability to withstand highest stress at its moment of yield the process control of combination foaming with talc-filled PP shows that there is a wide σ_fM_ distribution particularly with parts with low mass.

All the mechanical test data presented in this research, except foaming of unfilled PP, follows a clear trend, with reduced weight and increased foams, the mechanical properties decrease. The experimental data with generally the strongest mechanical properties, the chemically foamed parts, does not have superior cell density but, in the case of both PPs tested, they do have the thickest skin thicknesses. The current research trend is to obtain foamed polymer samples with the highest cell density but in this research, it can be observed that cell density has little influence on mechanical strength, in comparison to skin thickness; which has a major weighting on the tensile and flexural properties. Shish-kebabs show highly superior strength and modulus, but are only able to form on the skin layer due to the weak shear and fast solidification [31]. However, when the skin begins to reduce in size, then this can lead to reduced mechanical strength.

The simple model results in Figure 9 are again, under predictions of the actual results, apart from the highest weight-saving for the talc-filled PP using the hybrid foaming method. The conclusion for this is the same as previously mentioned for the tensile tests; the skin thickness used in the equations is inaccurate and it is recommended for future research that this is formulated to achieve more accurate results.

## 4. Conclusions

Two grades of Copolymer PP, unfilled and talc-filled, were foamed through various techniques to characterize their mechanical properties and link the existing modeling technique to the cellular structures. The novel use of combining two discreet foaming mechanisms of chemical and physical foaming to create a hybrid foaming process was used to compare conventional IM against different process variations of low-pressure FIM techniques. The additives within the chemical foaming agent, C_6_H_7_NaO_7_ and CaCO_3_, along with the talc-filled PP, have shown improved cellular structures. The modulus of elasticity (E) and ultimate tensile strength (S_u_) for both PP blends, exhibited the strongest values when foamed through CBAs alone. Meanwhile the lowest mechanical tensile strength was exhibited always through the hybrid method of both the chemical and physical blowing agents. Flexural strength follows a similar pattern to that of tensile data; that the CBA produces the strongest foamed parts while the hybrid methods are always the weakest. The smallest cell diameters, along with high cell density and superior homogenous cell structure, was found in the PP parts produced in combination with the hybrid foaming configuration. Part consistency (part weight and mechanical strength consistency) followed a clear trend that with increased foaming (higher weight savings), the less consistent the parts were. This was clear with all the foaming methods but prominent when the hybrid foaming configuration is used.

The results presented in this paper have been linked with previously published skin theory models to show how the mechanical properties of FIM parts are proportional to the skin thickness of cellular structure. The predicted results showed similar trends to that of the experimental results, but future work involves improving the accuracy of the model.

## Figures and Tables

**Table 1:** Input processing data used for unfilled PP.

Experimental Number	CBA (%)	SCF (grams)	Shot Volume (cm³)	Weight (grams)	Weight Reduction cf. Experiment No. 0 (%)
0	0	0.000	40.1	23.1 ± 0.03	0.00
1	1	0.000	28.3	21.3 ± 0.07	7.5 ± 0.42
2	2	0.000	27.4	21.1 ± 0.16	8.3 ± 0.81
3	5	0.000	26.4	20.4 ± 0.12	11.4 ± 0.64
4	0	0.134	26.7	21.2 ± 0.16	8.1 ± 0.81
5	0	0.144	26.2	21.1 ± 0.28	8.4 ± 1.34
6	0	0.173	23.9	20.4 ± 1.46	11.6 ± 6.45
7	1	0.075	25.7	21.2 ± 0.69	8.1 ± 3.12
8	2	0.075	25.2	20.9 ± 1.19	9.5 ± 5.29
9	5	0.075	24.6	20.4 ± 1.70	11.4 ± 7.50

**Table 2:** Input processing data used for talc-filled PP.

Experimental Number	CBA (%)	SCF (grams)	Shot Volume (cm³)	Weight (grams)	Weight Reduction cf. Experiment No. 0 (%)
0	0	0.000	40.1	28.1 ± 0.03	0.00
1	1	0.000	26.4	25.0 ± 0.04	11.1 ± 0.24
2	2	0.000	26.3	24.6 ± 0.19	12.5 ± 0.77
3	5	0.000	25.0	23.6 ± 0.16	16.1 ± 0.66
4	0	0.085	25.2	25.0 ± 0.19	10.9 ± 0.77
5	0	0.096	24.3	24.7 ± 0.33	12.2 ± 1.27
6	0	0.118	23.4	23.7 ± 0.87	15.7 ± 3.19
7	1	0.076	26.0	24.9 ± 1.41	11.4 ± 5.12
8	2	0.076	25.3	24.5 ± 0.97	12.9 ± 3.55
9	5	0.076	24.1	23.5 ± 1.52	16.4 ± 5.50

**Table 3:** Other major machine input data.

Input Data	Unfilled PP	Talc-Filled PP
Barrel Temps. Profile (°C)	200-190-190-180	220–210–210–190
Back Pressure (MPa)	18.5	14.5
Mold Clamp Force (kN)	1000	1000
Injection Speed (mm/s)	150	150
Mold Temp. (°C)	23	23

**Table 4:** Microstructure data.

	Unfilled PP			Talc-Filled PP		
Experimental Number	Avg. Cell Diameter (µm)	Cell Density (cells/cm³)	Wall Thickness (µm)	Avg. Cell Diameter (µm)	Cell Density (cells/cm³)	Wall Thickness (µm)
1	72.5	1.2 × 10^6^	765.4	166.3	1.6 × 10^5^	926.3
2	60.8	2.4 × 10^6^	601.8	135.1	3.1 × 10^5^	772.3
3	65.5	2.7 × 10^6^	612.8	78.7	1.5 × 10^6^	677.8
4	349.9	3.6 × 10^3^	393.3	158.9	1.8 × 10^5^	469.9
5	324.1	7.0 × 10^3^	352.5	138.5	2.5 × 10^5^	428.8
6	357.6	7.9 × 10^3^	302.5	123.8	4.3 × 10^5^	44.2
7	41.8	1.0 × 10^7^	431.0	91.5	7.6 × 10^5^	414.5
8	32.6	1.1× 10^7^	323.0	73.6	1.7 × 10^6^	91.1
9	33.2	1.1× 10^7^	298.8	54.1	3.6 × 10^6^	41.2
